# Effects of mining chemicals on fish: exposure to tailings containing Lilaflot D817M induces CYP1A transcription in Atlantic salmon smolt

**DOI:** 10.1186/s13104-015-1342-2

**Published:** 2015-08-29

**Authors:** Pål A. Olsvik, Henning A. Urke, Tom O. Nilsen, John B. Ulvund, Torstein Kristensen

**Affiliations:** National Institute of Nutrition and Seafood Research, Nordnesboder 1-2, 5005 Bergen, Norway; Norwegian Institute of Water Research, 7486 Trondheim, Norway; INAQ AS, 7462 Trondheim, Norway; UNI Research, 5006 Bergen, Norway; Faculty of Biosciences and Aquaculture, University of Nordland, 8049 Bodø, Norway

**Keywords:** Mining activity, Atlantic salmon, Flotation chemicals, Lilaflot D817M, Transcriptional responses

## Abstract

**Background:**

Mine tailings, containing metals and production chemicals such as flotation chemicals and flocculants, may pose an environmental threat to aquatic organisms living in downstream ecosystems. The aim of this work was to study to which degree Lilaflot D817M, a flotation chemical extensively used by the mining industry, represents a hazard for migrating salmon in rivers affected by mining activity. Smoltifying Atlantic salmon were exposed to four concentrations of iron-ore mine tailings containing residual Lilaflot D817M [water versus tailing volumes of 0.002 (Low), 0.004 (Medium), 0.013 (High) and 0.04 (Max)]. After 96 h of exposure, gill and liver tissues were harvested for transcriptional responses. Target genes included markers for oxidative stress, detoxification, apoptosis and DNA repair, cell signaling and growth.

**Results:**

Of the 16 evaluated markers, significant transcriptional responses of exposure to tailings enriched with Lilaflot D817M were observed for CYP1A, HSP70 and HMOX1 in liver tissue and CYP1A in gill tissue. The significant induction of CYP1A in both liver and gills suggest that the flotation chemical is taken up by the fish and activates cytochrome P450 detoxification via phase I biotransformation in the cells.

**Conclusions:**

The overall weak transcriptional responses to short-term exposure to Lilaflot D817M-containing iron-ore tailings suggest that the mining chemical has relatively low toxic effect on fish. The underlying mechanisms behind the observed CYP1A induction should be studied further.

## Background

Mining activity typically generates large amounts of tailings, crushed rock leftovers after extraction of minerals ranging from coarse sands down to a powder consistency [1]. Tailings from mine activity may, in addition to metals, contain considerable amounts of production chemicals such as flotation chemicals and flocculants [2, 3]. Disposal of tailings represents one of the main environmental problems generated by mining activity. In Norway, Canada and several other countries, the fine-grained tailings were traditionally deposited in artificial dams or natural lakes [4]. Due to the proximity to the ocean of many mines, sea disposal has historically been used and is currently considered as discharge points for several new mines in Norway [3]. Some of these fjords are deemed important for migratory Atlantic salmon (*Salmo salar*), raising concerns that mining chemicals might negatively affect local populations. Of special concern are seaward-bound juvenile fish, which are especially vulnerable during the physiologically demanding smoltification stage when they are pre-adapting to a marine environment [5]. In general, very little is known about the effects of these mining production chemicals on marine wildlife.

One of the flotation chemicals extensively used by the mining industry to increase sedimentation of suspended solids in water in Norway is Lilaflot D817M [4], with substantial releases into the Bøkfjorden and Ranfjorden areas in Northern Norway. This lipid-soluble chemical has a slow turnover in biological systems, and may bioaccumulate in exposed animals [6]. Because of its low water solubility, most of the deposited Lilaflot D817M will be bound to sediments, making bottom-dwelling organisms especially vulnerable for long-term effects [4]. In Bøkfjorden, where about 639 metric tons of Lilaflot D817M were released into the fjord between 1981 and 1997 by Sydvaranger Gruver AS, detectable amounts of Lilaflot D817M was found in fjord sediments 12 years after disposal was terminated [7]. The main chemical substances in Lilaflot D817M are *N*-(3-(tridecyloxy)propyl)-1,3-propane diamine (60–80 %) and *N*-(3-(tridecyloxy)propyl)-1,3-propane diamine acetate (20–40 %), with the first substance considered the most biologically active compound. According to the US Environmental Protection Agency [6], long-chain substituted propanediamines, the chemical group these compounds belongs to, are considered to be toxic to aquatic organisms, with observed lethality for plankton and fish at concentrations ranging from 0.75 to 170 µg L^−1^. Using elutriate made from tailings containing 56 mg kg^−1^ d.w., Berge et al. [8] observed acute toxic effects on microalgae (*Skeletonema costatum*) and crustacean (*Acartia tonsa*). Half this concentration resulted in effects on behavior and mortality in the polychaeta lugworm (*Arenicola marina*). In fish, the experiment indicated a 96-h LC50 value for turbot (*Scophthalmus maximus*) of 177 mg kg^−1^ with Lilaflot D817M [8]. According to our knowledge, no information exists on the molecular effects of Lilaflot D817M in fish or any other organisms.

The aim of this work was to use transcriptional responses to study the effects of mine tailings containing Lilaflot D817M on juvenile Atlantic salmon after exposure in brackish water, as part of an evaluation of the environmental impact of Lilaflot D817M released in the Ranfjorden area. Smolts were exposed to four concentrations of Lilaflot D817M for 96 h, and transcriptional responses in liver and gills compared to untreated controls. Based on known effects of the toxic components of Lilaflot D817M, a set of markers for potentially affected mechanisms were selected for transcriptional evaluation. These markers included genes known to respond to oxidative stress, detoxification, apoptosis and DNA repair, and growth.

## Results and discussion

### Exposure and survival

No experimental fish died, and no abnormal behavior was observed during the experiment. There was no significant size difference between the five groups of fish at sampling. Ingestion of particles and subsequent uptake through the intestine cannot be ruled out as an additional route of exposure. However, fish were not fed during the experiment and the low salinity during exposure should not cause drinking of seawater for ionoregulatory purposes.

### Water chemistry

Salinity was close to nominal values in all exposure groups, and pH increased slightly with increasing exposure (Table 1). Turbidity increased in a dose dependent fashion to a level of very low visibility in the High and Max groups (0.0013 and 0.04 water versus tailing volumes). The temperature was stable in all treatments during the experiment (Table 1). The mine tailings had a pH of 7.7 and suspended solids were 406 mg L^−1^. Mean particle diameter was 65.6 µm, while median was 37.6 µm. None of the 6 alkyletheramines/alkyletherdiamines were detected in the aqueous phase after 3 days of sedimentation (detection limit: 0.10 µg L^−1^), and the sum value was therefore assigned as <0.6 µg L^−1^. In the solid phase, the sum value was between 5.9 and 6.6 mg kg^−1^ for the duplicate analyses, and all measurements were above the detection limit. C13 di was the dominant constituent, with levels ranging from 3.7 to 4.1 mg kg^−1^.

**Table 1:** Water chemistry and temperature measurements in experimental tanks

Treatment group	Salinity (PSU)	pH	Turbidity (FNU)	Temperature (°C)
Control	5.5 ± 0.3	7.35 ± 0.03	0.4 ± 0.3	6.4 ± 0.7
Low	5.2 ± 0.6	7.34 ± 0.05	1.9 ± 0.5	6.4 ± 0.7
Medium	5.1 ± 0.7	7.35 ± 0.03	4.2 ± 0.7	6.4 ± 0.7
High	5.1 ± 0.6	7.42 ± 0.03	12.8 ± 1.8	6.3 ± 0.7
Max	5.1 ± 0.6	7.48 ± 0.02	36.7 ± 6.5	6.4 ± 0.6

Values are mean ± SD, N = 4 for salinity, pH and turbidity, and N = 192 for temperature

### Transcriptional results

**Fig. 1:**
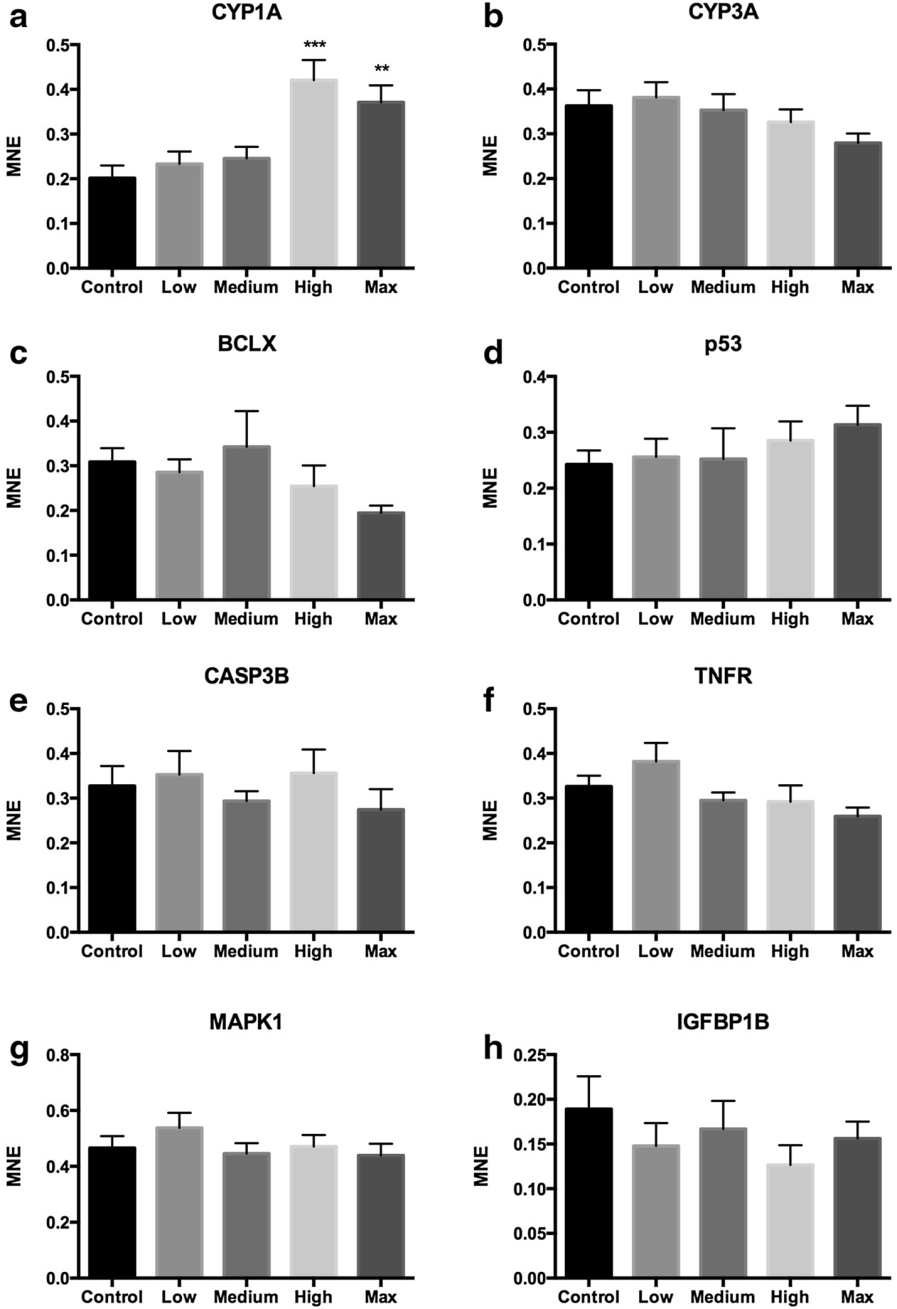
Detoxification, apoptosis and DNA repair, cell signaling, and growth markers in liver tissue of Atlantic salmon smolts exposed to tailings containing Lilaflot D817M. **a** CYP1A, **b** CYP3A, **c** BCLX, **d** P53, **e** CASP3B, **f** TNFR, **g** MAPK1 and **h** IGFBP1B. Values are given as mean ± SEM. *MNE* mean normalized expression. Control, High, Max: n = 8. Low, Medium: n = 7

**Fig. 2:**
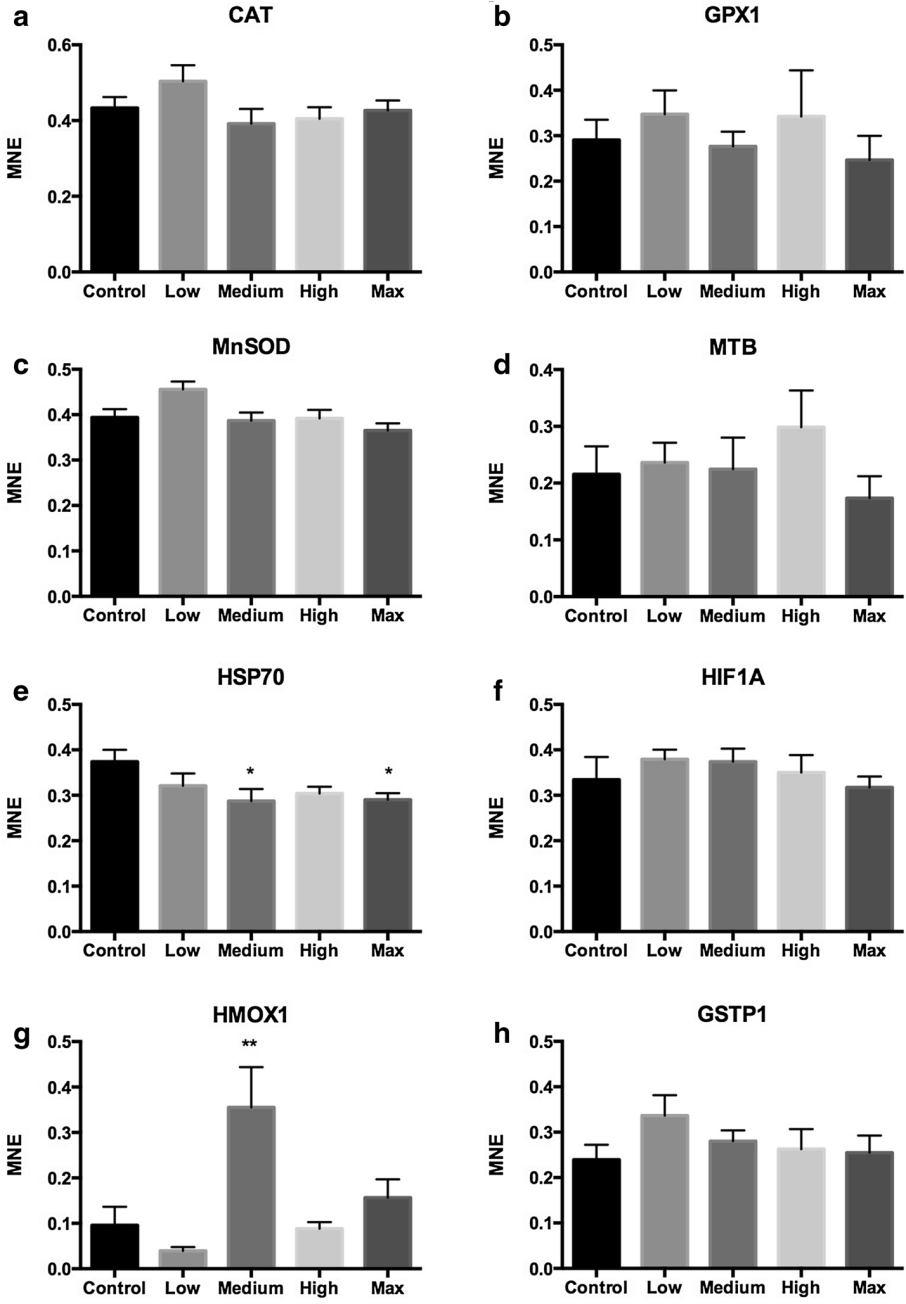
Oxidative stress markers in liver tissue of Atlantic salmon smolts exposed to tailings containing Lilaflot D817M. **a** CAT, **b** GPX1, **c** Mn SOD (SOD2), **d** MTB, **e** HSP70, **f** HIF1A, **g** HMOX1 and **h** GSTP1. Values are given as mean ± SEM. *MNE* mean normalized expression. Control, High, Max: n = 8. Low, Medium: n = 7

In general, the RT-qPCR data revealed few transcriptional responses of exposure to tailings containing Lilaflot D817M in liver tissue of juvenile Atlantic salmon (Figs. 1, 2). The most distinct response was a significant up-regulation of CYP1A in the two highest exposure groups. CYP1A was 2.1-fold up-regulated in liver of the High-exposure fish group (Fig. 1a, one-way ANOVA, P < 0.001), and 1.8-fold up-regulated in the Max exposure fish group compared to the control (P < 0.01). HSP70, on the other hand, was weakly but significantly down-regulated in the Medium and Max exposure fish groups compared to the control (Fig. 2e, 1.3-fold, P < 0.05). HMOX1 showed a surprising up-regulation in the Medium exposure group only (Fig. 2g, 3.7-fold, P < 0.01). For most of the oxidative stress markers, exposure to tailings containing Lilaflot D817M gave no effects, suggesting that the chemical has a low ability to induce oxidative stress. Our results showed no transcriptional responses on the selected markers for effects on mechanisms related to apoptosis, DNA repair and growth.

**Fig. 3:**
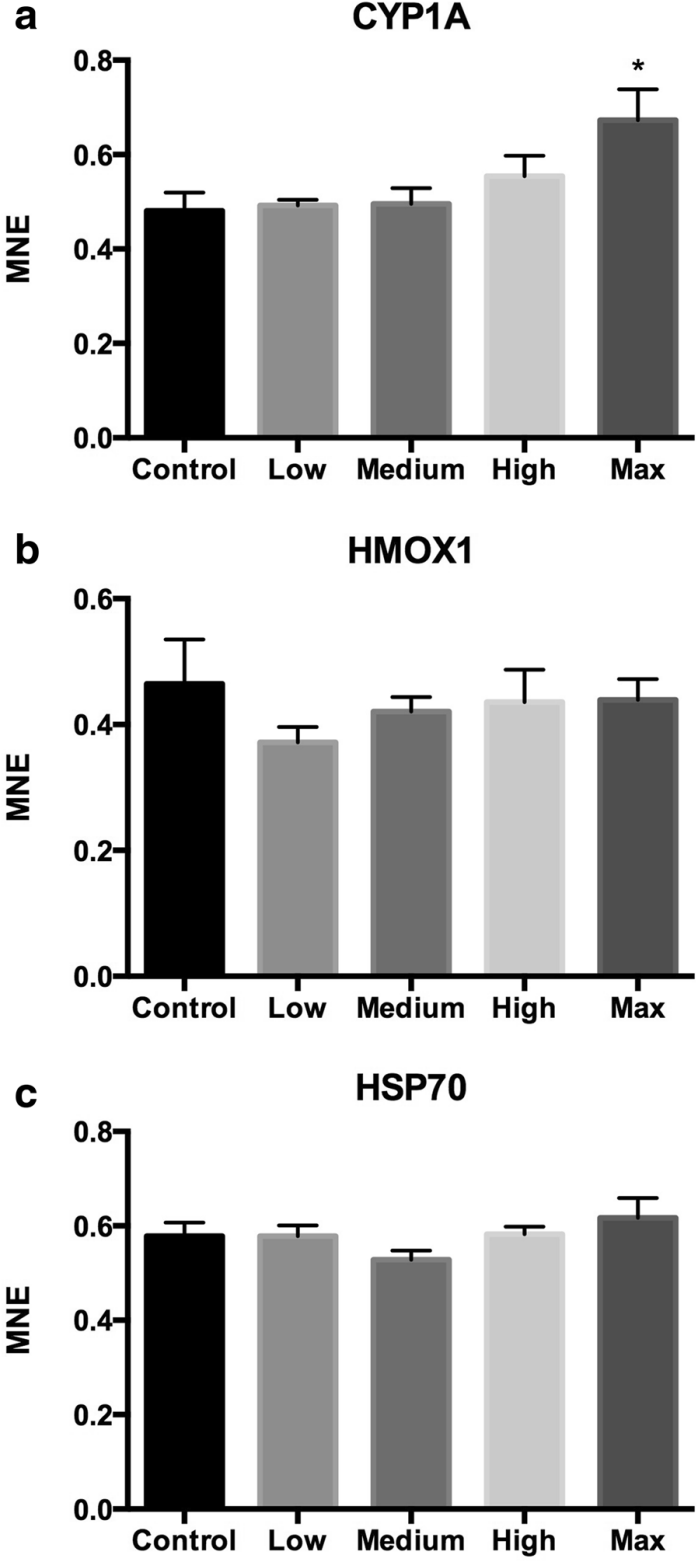
Gill tissue responses to tailings containing Lilaflot D817M exposure in Atlantic salmon smolts. **a** CYP1A, **b** HMOX1 and **c** HSP70. Values are given as mean ± SEM. *MNE* mean normalized expression. Control, Medium: n = 7. Low, High, Max: n = 8

In order to confirm the finding for CYP1A, and since we hypothesized that Lilaflot D817M exposure might affect gill physiology and ion regulation, the three gene transcripts that showed significant response in the liver were also quantified in gill tissue (Fig. 3). In line with the result from liver, CYP1A was significantly up-regulated in gills of fish from the Max group (Fig. 3a, 1.4-fold, one-way ANOVA, P < 0.05). HSP70 and HMOX1 were not differentially expressed in gill tissue.

Expression of CYP1A is regulated via the aryl hydrocarbon receptor [9]. The underlying mechanisms behind the observed transcriptional induction of CYP1A in liver and gill tissue are unknown. However, it is reasonable to hypothesize that bioactive components in Lilaflot D817M are oxidized by phase I biotransformation reactions and detoxified via this pathway. A number of xenobiotics as well as endogenous compounds are metabolized by CYP1A in fish [9]. Not only organic pollutants, but also heavy metals such as Cd, Cu and Hg have the ability to affect CYP1A transcription in mammals and fish [10–12]. In this experiment we used natural tailings as a source of Lilaflot D817M. The observed induction of CYP1A transcription may thus potentially rely on other factors than the flotation chemical itself. No other mine operation processing chemicals are however deposited in the tailings. The iron ore being mined in the region also contains very low levels of heavy metals that may affect CYP1A expression [13]. Iron itself, to our knowledge, is not known to be able to induce CYP1A transcription in fish. In an attempt to document possible effects of Lilaflot D817M on gill ion regulation, a tightly regulated mechanism in Atlantic salmon smolt adapting to high-salinity seawater, accumulation of various heavy metals on gill epithelium was measured in the current experiment. These data show low levels of heavy metals that may potentially affect CYP1A transcription. Of the measured metals Al, Cu, Fe, Mn and Zn, only Al and Mn showed significant (P = 0.05, Tukey–Kramer HSD) accumulation in the MAX group, with a 4-fold and 1.2-fold increase in concentration, respectively (Kristensen, unpublished data). The resulting absolute accumulation level for Al (20 g/g d.w.) is below effect-concentrations documented for Atlantic salmon smolts [14, 15], and most likely due to particle adhesion to gills rather than Al binding due to the high pH of the water. Consequently, the observed CYP1A induction in tissues of smoltifying Atlantic salmon most likely rely on mechanisms related to detoxification of components in the flotation chemical.

**Fig. 4:**
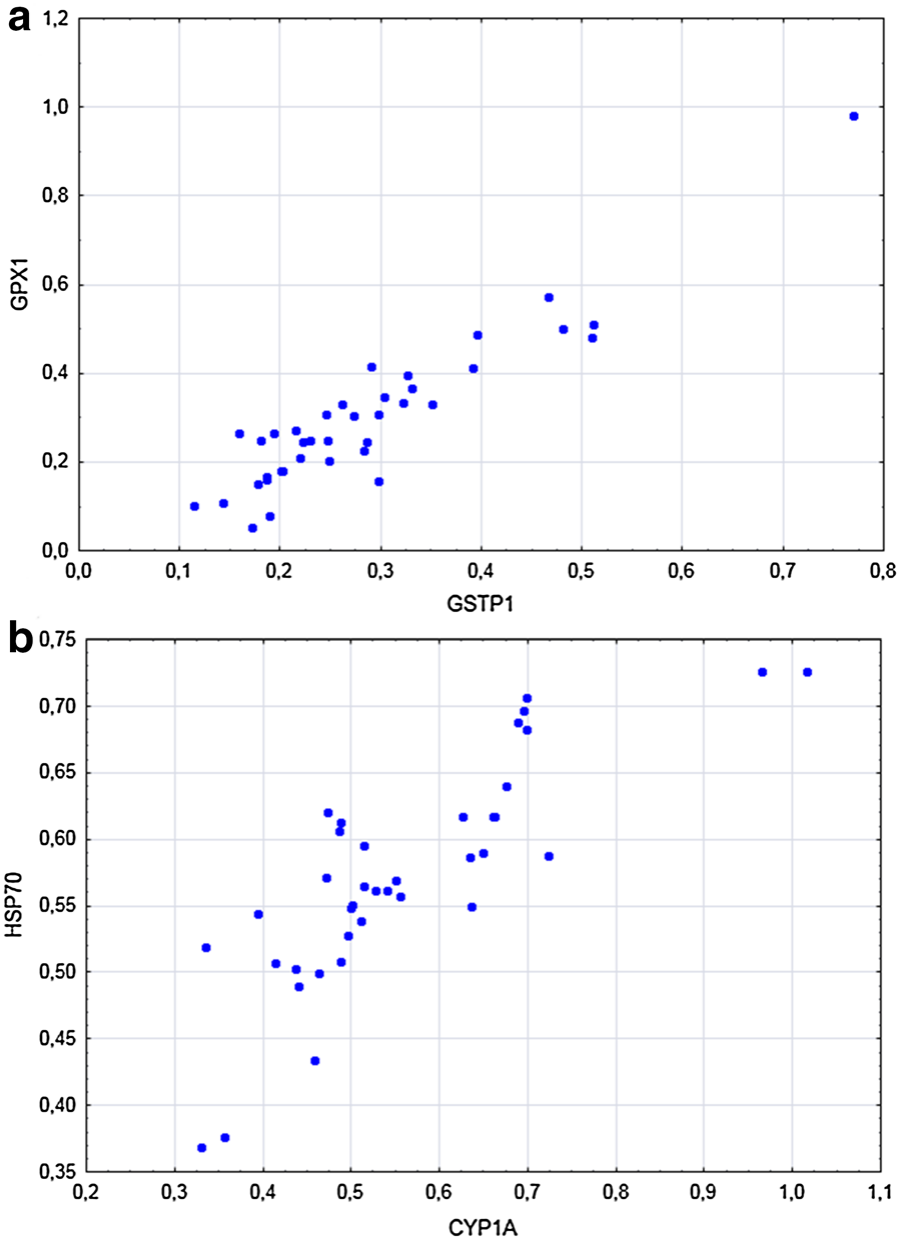
Correlation between **a** GSTP1 and GPX1 in liver tissue of Atlantic salmon smolts exposed to tailings containing Lilaflot D817M (Pearson’s correlation, r = 0.93) and **b** CYP1A and HSP70 in gill tissue of Atlantic salmon exposed to tailings containing Lilaflot D817M (Pearson’s correlation, r = 0.82)

Correlation analysis was conducted to search for effect of treatment group (dose–response effects) using a different statistical method, and to search for co-regulation of gene transcripts that may possible add to the mechanistic understanding of the impact of the mining chemical. In liver tissue (Figs. 1, 2) there was a positive treatment group correlation for CYP1A (Pearson’s correlation analysis, r = 0.58), while there were negative treatment group correlations for CYP3A (r = −0.36) and HSP70 (r = −0.47). Many of the evaluated gene transcripts showed a relatively strong co-regulation in liver, as to be expected since many of them belong to the same pathways. This was true especially for the oxidative stress markers. The strongest observed correlation in liver tissue was between the GSTP1 and GPX1 transcripts (Fig. 4a, Pearson’s correlation analysis, r = 0.93). This response is most likely not due to the chemical exposure, but rather a result of intrinsic mechanisms in the cells. Of the three genes evaluated in gill tissue, no significant treatment group correlations were seen. There was a strong positive correlation between CYP1A and HSP70 expression (Fig. 4b, Pearson’s correlation analysis, r = 0.82) in gill tissue. Although modest, the effect seen for HSP70 in liver tissue indicates that the exposure may have affected mechanisms linked to protein stability and the ubiquitin–proteasome pathway [16]. Collectively, these findings show that the flotation chemical, except for CYP1A, has relatively modest ability to affect the transcription of genes often responding to environmental contaminants.

## Conclusions

The current study shows that Lilaflot D817M-containing mine tailings from Rana Gruber, to 0.04 % of total water influx, can induce CYP1A transcription in Atlantic salmon smolt. The exact mechanisms behind this induction are unknown, but our results suggest that the flotation chemical to a certain degree can be taken up by the fish after waterborne exposure and transported to the gills and liver for cytochrome P450 detoxification via the phase I biotransformation system. Based on the lack of, or weak responses observed for gene transcripts easily induced by lipid-soluble environmental contaminants, our overall data indicate that the toxicity of the mine tailings on Atlantic salmon is only modest.

## Methods

### Experimental setup

Experimental conditions were chosen to simulate surface release of mine tailings to a fjord system at the time of smolt migration (May–June). Freshwater discharge data for River Ranelva (20–360 m^3^ s^−1^), the main contributing freshwater source in the fjord system, was used in combination with known tailings discharge (0.8 m^3^ s^−1^) to generate exposure regimes. Factors of water versus tailing volumes of 0.002 (Low), 0.004 (Medium), 0.013 (High) and 0.04 (Max) were used along with and a control group without added tailing. The exposures were conducted at 5 psu salinity, simulating the uppermost water levels where Atlantic salmon smolt migrate [17]. Fresh and seawater were mixed and aerated in a header tank (350 L) to nominally 5 psu before water was supplied by gravity to hexagonal fish tanks (150 L water volume) at a rate of 2 L min^−1^. Mine tailings were added from 20 L containers to each tank by a peristaltic pump. Fresh mine tailings were obtained from the nearby production plant twice during the experiment. Water flow and salinity, and tailings flow, was monitored minimum every 12th hour during the experiment. Water temperature (Table 1) was logged at 30 min intervals in all tanks during the experiment (HOBO Pendant Temperature/Light Data Logger, http://www.onsetcomp.com).

### Fish material and sampling

Atlantic salmon smolts of local Røssåga strain raised in the Bjerka live gene-bank facility were used in the experiment. The fish used for transcriptional analysis were in average weighing 29.6 ± 1.0 g and having a length of 14.8 ± 0.2 cm (N = 38), with no significant differences in weight between the groups at the time of tissue collection. Fish were randomly collected from a holding tank and transferred to the exposure tanks (N = 20) 24 h before start of exposure. The fish were not fed during the experiment. The fish were closely monitored for mortalities and abnormal behavior during the experiment. At the final sampling time after 96 h of exposure, eight fish from each treatment group were netted from the tanks and sampled within 5 min for transcriptional analysis (N = 40). Fish were killed by a sharp blow to the head, and tissues (2nd gill arch and liver) dissected out and transferred to 1.8 mL cryovials containing 1 mL RNA-later (Ambion Inc.; http://www.ambion.com), stored at 4 °C for 24 h, then in liquid nitrogen before being shipped on dry ice for analysis.

### Chemical analysis

Water samples from all tanks were collected daily and analyzed for pH, salinity and turbidity (Table 1). The mine tailings were analyzed for suspended material content, pH, and size distribution (Coulter LS 230, laser diffraction). Alkyletheramines and alkyldietheramines (C12–C14) were measured in the aqueous phase (six replicates) and solid phase (two replicates) after 3 days of sedimentation (Table 2). Alkyletheramines and alkyldietheramines from tailings in liquid and solid phase were measured by LCMS in MRM-mode with a C18-column, methanol as mobile-phase gradient and ammoniumtrifluoroacetate/ammoniumacetate buffer (pH 4.5) in both phases (AkzoNobel, 12 AC 0269).

**Table 2:** Chemical analysis of alkyletheramines and alkyletherdiamines (C12–C14) in mine tailings used in the experiment

C12 mono	C13 mono	C14 mono	C12 di	C13 di	C14 di	Sum
0.12	0.39	0.066	1.03	3.7	0.62	5.9
0.14	0.43	0.071	1.20	4.1	0.70	6.6
*0.13*	*0.41*	*0.070*	*1.10*	*3.9*	*0.66*	*6.3*

Results from solid phase calculated as mg kg^−1^ dry solid (duplicate measurements, mean values in italics)

### RNA isolation

Tissues from Atlantic salmon were homogenized with the Precellys 24 homogenizer by using ceramic beads CK28 (Bertin Technologies, Montigny-le-Bretonneux, France). Total RNA was extracted using the BioRobot EZ1 and RNA Tissue Mini Kit (Qiagen, Hilden, Germany) and treated with DNase according to the manufacturer’s instructions and eluted in 50 μL RNase-free MilliQ H_2_O. The RNA was then stored at −80 °C before further processing. RNA quality and integrity were assessed with the NanoDrop ND-1000 UV–Vis Spectrophotometer (NanoDrop Technologies, Wilmington, DE, USA) and the Agilent 2100 Bioanalyzer (Agilent Technologies, Palo Alto, CA, USA). The 260/280 and 260/230 nm ratios in liver were 2.11 ± 0.01 and 2.22 ± 0.01, respectively (N = 38, mean ± SEM). The RNA 6000 Nano LabChip kit (Agilent Technologies, Palo Alto, CA, USA) was used to evaluate the RNA integrity of the samples. The RNA integrity number (RIN) was 9.3 ± 0.1 (N = 12) in liver (mean ± SEM).

### Quantitative real-time RT-qPCR

PCR primer sequences used for quantification of the transcriptional levels of the evaluated genes are shown in Table 3. Sixteen target genes and three reference genes were quantified with RT-qPCR. BLASTX or BLASTN was used to determine PCR assay specificity. The reaction specificity of each assay was verified by observing a single peak in the melting curve. The RT-qPCR work was conducted according to the MIQE guidelines [18].

**Table 3:** PCR primers, accession or contig numbers, amplicon sizes and PCR efficiencies

Gene product	Gene name	Marker for	Accession no.	Forward primer	Reverse primer	Amplicon size (bp)	PCR efficiency ^liver/gills^
Catalase	CAT	Oxidative stress	BT059457	CCCAAGTCTTCATCCAGAAACG	CGTGGGCTCAGTGTTGTTGA	123	2.03
Glutathione peroxidase 1	GPX1	Oxidative stress	DW566563	GCCCACCCCTTGTTTGTGTA	AGACAGGGCTCCACATGATGA	103	1.96
Manganese superoxide dismutase	MNSOD	Oxidative stress	DY718412	GTTTCTCTCCAGCCTGCTCTAAG	CCGCTCTCCTTGTCGAAGC	227	1.87
Heme oxygenase 1	HMOX1	Iron metabolism/oxidative stress	BG936101	AGCAGATTAAAGCTGTAACCAAGGA	GCCAGCATCAGCTCAGTGTTC	64	2.07/1.75
Heat shock protein 70	HSP70	Protein folding/oxidative stress	BG933934	CCCCTGTCCCTGGGTATTG	CACCAGGCTGGTTGTCTGAGT	121	2.04/2.06
Metallothionein B	MTB	Stress/oxidative stress	CK990996	TGAATAAAGAAGCGCGATCAAA	CTGGTGCATGCGCAGTTG	111	1.87
Hypoxia-inducible factor 1A	HIF1A	Oxidative stress	DY708816	CCACCTCATGAAGACCCATCA	TCTCCACCCACACAAAGCCT	101	1.94
Cytochrome P450 1A	CYP1A	Detoxification	AF364076	TGGAGATCTTCCGGCACTCT	CAGGTGTCCTTGGGAATGGA	101	2.06/1.92
Cytochrome P450 3A	CYP3A	Detoxification	DQ361036	ACTAGAGAGGGTCGCCAAGA	TACTGAACCGCTCTGGTTTG	146	2.00
Glutathione S-transferase P1	GSTP1	Detoxification/oxidative stress	BQ036247	ATTTTGGGACGGGCTGACA	CCTGGTGCTCTGCTCCAGTT	81	2.11
Tumor suppressor p53	P53	DNA damage	BT058777	CTCGCCAGACCTGAACAAGTT	ATAGATGGCCAGGGCTCGTA	112	2.34
Apoptosis regulator Bcl-X	BCLX	DNA damage/apoptosis	NM_001141086	GCCTGGACGCAGTGAAAGAG	GGACGGCGTGATGTGTAGCT	107	1.99
Insulin-like growth factor binding protein 1B	IGFBP1B	Anti-growth	AY662657	GAGGACCAGGGACAAGAGAAAGT	GCACCCTCATTTTTGGTGTCA	101	1.98
Mitogen-activated kinase 1	MAPK1	MAP kinase activity	EF101948	TCAATCTGGAGAAGGAGCTCGTA	CTACCTGCCGTAGCTCTTCGAT	51	1.81
Caspase 3B	CASP3B	Apoptosis	DQ008069	AGCCGATTCGGTGTTAAAAGG	CCGGAGGCTTAGCGTCTACTT	107	2.08
Tumor necrosis factor receptor superfamily 1A	TNFRSF1A	Infammation/apoptosis	NM_001141773	AAGACCTGCCTCCGTTGTACA	CTGAGGCACTCCCGTGTTTC	140	1.96
B-actin	ACTB	Reference gene	BG933897	CCAAAGCCAACAGGGAGAA	AGGGACAACACTGCCTGGAT	92	1.94/1.85
Eukaryotic translation elongation factor 1 alpha B	EEF1AB	Reference gene	BG933853	TGCCCCTCCAGGATGTCTAC	CACGGCCCACAGGTACTG	59	2.00
Ribosomal protein L13	RPL13	Reference gene	NM_001141291	CCAATGTACAGCGCCTGAAA	CGTGGCCATCTTGAGTTCCT	110	2.02/2.05
60S ribosomal protein L40	UBA52	Reference gene	GO054675	GATCTTCGCTGGCAAACAACT	CGAAGACGCAGCACAAGATG	93	/2.12

Real-time RT-qPCR was conducted as previously described by Olsvik et al. [19]. Briefly, a two-step real-time RT-PCR protocol was used to quantify the transcriptional levels of the selected genes. The RT reactions were run in duplicate using 96-well reaction plates with the GeneAmp PCR 9700 (Applied Biosystems, Foster City, CA, USA) with TaqMan Reverse Transcription Reagent containing Multiscribe Reverse Transcriptase (50 U µL^−1^) (Applied Biosystems, Foster City, CA, USA). Two-fold serial dilutions of total RNA were made for efficiency calculations. Six serial dilutions (1000–31 ng RNA) in triplicates were analyzed in separate sample wells. Total RNA input was 500 ng in each reaction for all genes. No template controls (ntc) and RT-controls (no amplification controls, nac) were run for quality assessment for each PCR assay.

Reverse transcription was performed at 48 °C for 60 min by using oligo dT primers (2.5 μM) for all genes in 50 µL total volume. The final concentration of the other chemicals in each RT reaction was: MgCl_2_ (5.5 mM), dNTP (500 mM of each), 10× TaqMan RT buffer (1×), RNase inhibitor (0.4 U µL^−1^) and Multiscribe reverse transcriptase (1.67 U μL^−1^) (Applied Biosystems). Twofold diluted cDNA was transferred to 384-well reaction plates and the qPCR run in 10 μL reactions on the LightCycler 480 Real-Time PCR System (Roche Applied Sciences, Basel, Switzerland). Real-time PCR was performed using SYBR Green Master Mix (LightCycler 480 SYBR Green master mix kit, Roche Applied Sciences), which contains FastStart DNA polymerase and gene-specific primers (500 nM of each). PCR was achieved with a 5 min activation and denaturizing step at 95 °C, followed by 45 cycles of a 10 s denaturing step at 95 °C, a 10 s annealing step at 60 °C and a 10 s synthesis step at 72 °C. Target gene mean normalized expression (MNE) was determined using a normalization factor based upon ACTB, EEF1AB, and RPL13 as calculated by the geNorm software [20]. The geNorm stability index M was less than 0.42 for all reference genes.

### Data analysis

The GraphPad Prism 5.0 software (GraphPad Software, Inc., San Diego, CA, USA) was used for statistical analyses of the gene expression data. One-way ANOVA with Dunnett’s multiple comparison test (for comparison to the control) and Pearson’s correlation analysis were used to compare the transcriptional levels of the examined genes between the experimental groups. In case the Bartlett’s test showed that the variances differed, the mean normalized expression (MNE) data were log-transformed before ANOVA analysis. ROUT (Q = 1.000 %) outlier test was used to screen for outliers. Correlation analysis was performed using the program Statistica 8.0 (Statsoft Inc., Tulsa, USA). A significance level of P < 0.05 was used for all tests.

## References

[1] Luoma SN, Rainbow PS (2011). Metal contamination in aquatic environments.

[2] Liber K, Weber L, Levesque C (2005). Sublethal toxicity of two wastewater treatment polymers to lake trout fry (*Salvelinus namaycush*). Chemosphere.

[3] Skei JM, Syvitski JPM. Natural flocculation of mineral particles in seawater—influence on mine tailings sea disposal and particle dispersal. Mineralproduksjon. 2013;3:A1–A10. http://mineralproduksjon.no/wp-content/uploads/2014/03/mp3-00-hele.pdf.

[4] Klif. Bergverk og avgangsdeponering, Status, miljøutfordringer og kunnskapsbehov. Klima og Forurensnings Direktoratet (Klif) TA 2715;2010 **(In Norwegian, English summary)**.

[5] Thorstad EB, Uglem I, Arechavala-Lopez P, Økland F, Finstad B (2011). Low survival of hatchery-released Atlantic salmon smolts during initial river and fjord migration. Boreal Environ Res.

[6] EPA. Fatty nitrogen derived amines category high production volume (HPV) chemicals challenge. Assessment of data availability and test plan. Prepared for American Chemistry Council’s Fatty Nitrogen Derivatives Panel Amines Task Group. Prepared by Toxicology/Regulatory Services, Inc. 201-14171a. 2003. http://www.epa.gov/hpv/pubs/summaries/amines/c14171rt1.pdf.

[7] Berge JA. Gruvekjemikalier i sedimentene i sjøområdene utenfor Kirkenes i 2009. Niva rapport L.NR. 5860-2009. 2009 **(In Norwegian, English summary)**.

[8] Berge JA. Giftighetstester med flotasjonskjemikaliet Lilaflot D817M. Effekter på alger, børstemark, krepsdyr of fisk. NIVA rapport L.NR. 6044-2010 **(In Norwegian, English summary)**.

[9] Schlenk D, Celander M, Gallagher EP, George S, James M, Kullman SW, Hurk PVD, Willett K, Giulio RT, Hinton DE (2008). Biotransformation in fishes. The toxicology of fishes.

[10] Tully DB, Collins BJ, Overstreet JD, Smith CS, Dinse GE, Mumtaz MM, Chapin R (2000). Effects of arsenic, cadmium, chromium, and lead on gene expression regulated by a battery of 13 different promoters in recombinant HepG2 cells. Toxicol Appl Pharmacol.

[11] Korashy HM, Ei-Kadi AOS (2008). The role of redox-sensitive transcription factors NF-kappa B and AP-1 in the modulation of the Cyp1a1 gene by mercury, lead, and copper. Free Rad Biol Med.

[12] Søfteland L, Holen E, Olsvik PA (2010). Toxicological application of primary hepatocyte cell cultures of Atlantic cod (*Gadus morhua*)—effects of BNF, PCDD and Cd. Comp Biochem Physiol C-Toxicol Pharmacol.

[13] Rana Gruber. http://www.ranagruber.no/index.php?id=50&L=1.

[14] Kroglund F, Finstad B, Stefansson SO, Nilsen TO, Kristensen T, Rosseland BO, Teien HC, Salbu B (2007). Exposure to moderate acid water and aluminum reduces Atlantic salmon post-smolt survival. Aquacult.

[15] Nilsen TO, Ebbesson LOE, Kverneland OG, Kroglund F, Finstad B, Stefansson SO (2010). Effects of acidic water and aluminum exposure on gill Na+, K+-ATPase alpha-subunit isoforms, enzyme activity, physiology and return rates in Atlantic salmon (*Salmo salar* L.). Aquat Toxicol.

[16] GeneCards Database. http://www.genecards.org.

[17] Plantalech Manel-La N, Thorstad EB, Davidsen JG, Økland F, Sivertsgård R, McKinley RS, Finstad B (2009). Vertical movements of Atlantic salmon post-smolts relative to measures of salinity and water temperature during the first phase of the marine migration. Fish Manag Ecol.

[18] Bustin SA, Benes V, Garson JA, Hellemans J, Huggett J, Kubista M, Mueller R, Nolan T, Pfaffl MW, Shipley GL, Vandesompele J, Wittwer CT (2009). The MIQE guidelines: minimum information for publication of quantitative real-time PCR experiments. Clin Chem.

[19] Olsvik PA, Lindgren M, Maage A (2013). Mercury contamination in deep-water fish: transcriptional responses in tusk (*Brosme brosme*) from a fjord gradient. Aquat Toxicol.

[20] Vandesompele J, De Preter K, Pattyn F, Poppe B, Van Roy N, De Paepe A, Speleman F (2002). Accurate normalization of real-time quantitative RT-PCR data by geometric averaging of multiple internal control genes. Genome Biol.

